# Supplementary material for: A fault-intrusion-tolerant system and deadline-aware algorithm for scheduling scientific workflow in the cloud
Source: PeerJ Comput Sci. 2021 Nov 2;7:e747. doi: 10.7717/peerj-cs.747 (PMC8576554; doi:10.7717/peerj-cs.747)
Supplement: Supplemental Information 6 [file peerj-cs-07-747-s006.zip › Myproject1.1/docs/org/cloudbus/cloudsim/core/CloudSimTags.html]

CloudSimTags


JavaScript is disabled on your browser.


- Overview
- Package
- Class
- Use
- Tree
- Deprecated
- Index
- Help

- Prev Class
- Next Class

- Frames
- No Frames

- All Classes

- Summary:
- Nested |
- Field |
- Constr |
- Method

- Detail:
- Field |
- Constr |
- Method


org.cloudbus.cloudsim.core

## Class CloudSimTags

- java.lang.Object
- - org.cloudbus.cloudsim.core.CloudSimTags

- ---

    

  ```
  public class CloudSimTags
  extends java.lang.Object
  ```

  Contains various static command tags that indicate a type of action that needs to be undertaken
  by CloudSim entities when they receive or send events. **NOTE:** To avoid conflicts with other
  tags, CloudSim reserves negative numbers, 0 - 299, and 9600.

  Since:
  :   CloudSim Toolkit 1.0

- - ### Field Summary

    Fields

    | Modifier and Type | Field and Description |
    | --- | --- |
    | `static int` | `ABRUPT_END_OF_SIMULATION` Denotes an abrupt end of simulation. |
    | `static int` | `CLOUDLET_CANCEL` Cancels a Cloudlet submitted in the CloudResource entity. |
    | `static int` | `CLOUDLET_MOVE` Moves a Cloudlet to another CloudResource entity. |
    | `static int` | `CLOUDLET_MOVE_ACK` Moves a Cloudlet to another CloudResource entity with an acknowledgement. |
    | `static int` | `CLOUDLET_PAUSE` Pauses a Cloudlet submitted in the CloudResource entity. |
    | `static int` | `CLOUDLET_PAUSE_ACK` Pauses a Cloudlet submitted in the CloudResource entity with an acknowledgement. |
    | `static int` | `CLOUDLET_RESUME` Resumes a Cloudlet submitted in the CloudResource entity. |
    | `static int` | `CLOUDLET_RESUME_ACK` Resumes a Cloudlet submitted in the CloudResource entity with an acknowledgement. |
    | `static int` | `CLOUDLET_RETURN` Denotes the return of a Cloudlet back to sender. |
    | `static int` | `CLOUDLET_STATUS` Denotes the status of a Cloudlet. |
    | `static int` | `CLOUDLET_SUBMIT` Denotes the submission of a Cloudlet. |
    | `static int` | `CLOUDLET_SUBMIT_ACK` Denotes the submission of a Cloudlet with an acknowledgement. |
    | `static int` | `DEFAULT_BAUD_RATE` Denotes the default baud rate for some CloudSim entities |
    | `static int` | `END_OF_SIMULATION` Denotes the end of simulation |
    | `static int` | `EXPERIMENT` Sends an Experiment object between UserEntity and Broker entity |
    | `static int` | `FALSE` Denotes boolean false in int value |
    | `static int` | `INFOPKT_RETURN` This tag is used to return the ping request back to sender |
    | `static int` | `INFOPKT_SUBMIT` This tag is used by an entity to send ping requests |
    | `static int` | `INSIGNIFICANT` Denotes insignificant simulation entity or time. |
    | `static int` | `Network_Event_DOWN` |
    | `static int` | `Network_Event_Host` |
    | `static int` | `Network_Event_send` |
    | `static int` | `Network_Event_UP` |
    | `static int` | `NextCycle` |
    | `static int` | `RECORD_STATISTICS` Denotes a request to record events for statistical purposes. |
    | `static int` | `REGISTER_REGIONAL_GIS` Denotes a request to register a CloudResource entity to a regional CloudInformationService (GIS) entity |
    | `static int` | `REGISTER_RESOURCE` Denotes a grid resource to be registered. |
    | `static int` | `REGISTER_RESOURCE_AR` Denotes a grid resource, that can support advance reservation, to be registered. |
    | `static int` | `REQUEST_REGIONAL_GIS` Denotes a request to get a list of other regional GIS entities from the system GIS entity |
    | `static int` | `RESOURCE_AR_LIST` Denotes a list of hostList that only support advance reservation. |
    | `static int` | `RESOURCE_CHARACTERISTICS` Denotes grid resource characteristics information. |
    | `static int` | `RESOURCE_CHARACTERISTICS_REQUEST` Denotes request for grid resource characteristics information. |
    | `static int` | `RESOURCE_DYNAMICS` Denotes grid resource allocation policy. |
    | `static int` | `RESOURCE_LIST` Denotes a list of all hostList, including the ones that can support advance reservation. |
    | `static int` | `RESOURCE_NUM_FREE_PE` Denotes a request to get the total number of free Processing Elements (PEs) of a resource. |
    | `static int` | `RESOURCE_NUM_PE` Denotes a request to get the total number of Processing Elements (PEs) of a resource. |
    | `static int` | `RESOURCE_Register` |
    | `static int` | `RETURN_ACC_STATISTICS_BY_CATEGORY` Denotes a request to send an Accumulator object based on category into an event scheduler. |
    | `static int` | `RETURN_STAT_LIST` Denotes a request to get a statistical list. |
    | `static double` | `SCHEDULE_NOW` Schedules an entity without any delay |
    | `static int` | `TRUE` Denotes boolean true in int value |
    | `static int` | `VM_BROKER_EVENT` Denotes an internal event generated in a Broker |
    | `static int` | `VM_CREATE` Denotes a request to create a new VM in a Datacentre With acknowledgement information sent by the Datacentre |
    | `static int` | `VM_CREATE_ACK` Denotes a request to create a new VM in a Datacentre With acknowledgement information sent by the Datacentre |
    | `static int` | `VM_DATA_ADD` Denotes an event to send a file from a user to a datacenter |
    | `static int` | `VM_DATA_ADD_ACK` Denotes an event to send a file from a user to a datacenter |
    | `static int` | `VM_DATA_DEL` Denotes an event to remove a file from a datacenter |
    | `static int` | `VM_DATA_DEL_ACK` Denotes an event to remove a file from a datacenter |
    | `static int` | `VM_DATACENTER_EVENT` Denotes an internal event generated in a PowerDatacenter |
    | `static int` | `VM_DESTROY` Denotes a request to destroy a new VM in a Datacentre |
    | `static int` | `VM_DESTROY_ACK` Denotes a request to destroy a new VM in a Datacentre |
    | `static int` | `VM_MIGRATE` Denotes a request to migrate a new VM in a Datacentre |
    | `static int` | `VM_MIGRATE_ACK` Denotes a request to migrate a new VM in a Datacentre With acknowledgement information sent by the Datacentre |
  - ### Method Summary

    - ### Methods inherited from class java.lang.Object

      `clone, equals, finalize, getClass, hashCode, notify, notifyAll, toString, wait, wait, wait`

- - ### Field Detail


    - #### TRUE

      ```
      public static final int TRUE
      ```

      Denotes boolean true in int value

      See Also:
      :   Constant Field Values


    - #### FALSE

      ```
      public static final int FALSE
      ```

      Denotes boolean false in int value

      See Also:
      :   Constant Field Values


    - #### DEFAULT\_BAUD\_RATE

      ```
      public static final int DEFAULT_BAUD_RATE
      ```

      Denotes the default baud rate for some CloudSim entities

      See Also:
      :   Constant Field Values


    - #### SCHEDULE\_NOW

      ```
      public static final double SCHEDULE_NOW
      ```

      Schedules an entity without any delay

      See Also:
      :   Constant Field Values


    - #### END\_OF\_SIMULATION

      ```
      public static final int END_OF_SIMULATION
      ```

      Denotes the end of simulation

      See Also:
      :   Constant Field Values


    - #### ABRUPT\_END\_OF\_SIMULATION

      ```
      public static final int ABRUPT_END_OF_SIMULATION
      ```

      Denotes an abrupt end of simulation. That is, one event of this type is enough for
      `CloudSimShutdown` to trigger the end of the simulation

      See Also:
      :   Constant Field Values


    - #### INSIGNIFICANT

      ```
      public static final int INSIGNIFICANT
      ```

      Denotes insignificant simulation entity or time. This tag will not be used for identification
      purposes.

      See Also:
      :   Constant Field Values


    - #### EXPERIMENT

      ```
      public static final int EXPERIMENT
      ```

      Sends an Experiment object between UserEntity and Broker entity

      See Also:
      :   Constant Field Values


    - #### REGISTER\_RESOURCE

      ```
      public static final int REGISTER_RESOURCE
      ```

      Denotes a grid resource to be registered. This tag is normally used between
      CloudInformationService and CloudResouce entity.

      See Also:
      :   Constant Field Values


    - #### REGISTER\_RESOURCE\_AR

      ```
      public static final int REGISTER_RESOURCE_AR
      ```

      Denotes a grid resource, that can support advance reservation, to be registered. This tag is
      normally used between CloudInformationService and CloudResouce entity.

      See Also:
      :   Constant Field Values


    - #### RESOURCE\_LIST

      ```
      public static final int RESOURCE_LIST
      ```

      Denotes a list of all hostList, including the ones that can support advance reservation. This
      tag is normally used between CloudInformationService and CloudSim entity.

      See Also:
      :   Constant Field Values


    - #### RESOURCE\_AR\_LIST

      ```
      public static final int RESOURCE_AR_LIST
      ```

      Denotes a list of hostList that only support advance reservation. This tag is normally used
      between CloudInformationService and CloudSim entity.

      See Also:
      :   Constant Field Values


    - #### RESOURCE\_CHARACTERISTICS

      ```
      public static final int RESOURCE_CHARACTERISTICS
      ```

      Denotes grid resource characteristics information. This tag is normally used between CloudSim
      and CloudResource entity.

      See Also:
      :   Constant Field Values


    - #### RESOURCE\_DYNAMICS

      ```
      public static final int RESOURCE_DYNAMICS
      ```

      Denotes grid resource allocation policy. This tag is normally used between CloudSim and
      CloudResource entity.

      See Also:
      :   Constant Field Values


    - #### RESOURCE\_NUM\_PE

      ```
      public static final int RESOURCE_NUM_PE
      ```

      Denotes a request to get the total number of Processing Elements (PEs) of a resource. This
      tag is normally used between CloudSim and CloudResource entity.

      See Also:
      :   Constant Field Values


    - #### RESOURCE\_NUM\_FREE\_PE

      ```
      public static final int RESOURCE_NUM_FREE_PE
      ```

      Denotes a request to get the total number of free Processing Elements (PEs) of a resource.
      This tag is normally used between CloudSim and CloudResource entity.

      See Also:
      :   Constant Field Values


    - #### RECORD\_STATISTICS

      ```
      public static final int RECORD_STATISTICS
      ```

      Denotes a request to record events for statistical purposes. This tag is normally used
      between CloudSim and CloudStatistics entity.

      See Also:
      :   Constant Field Values


    - #### RETURN\_STAT\_LIST

      ```
      public static final int RETURN_STAT_LIST
      ```

      Denotes a request to get a statistical list.

      See Also:
      :   Constant Field Values


    - #### RETURN\_ACC\_STATISTICS\_BY\_CATEGORY

      ```
      public static final int RETURN_ACC_STATISTICS_BY_CATEGORY
      ```

      Denotes a request to send an Accumulator object based on category into an event scheduler.
      This tag is normally used between ReportWriter and CloudStatistics entity.

      See Also:
      :   Constant Field Values


    - #### REGISTER\_REGIONAL\_GIS

      ```
      public static final int REGISTER_REGIONAL_GIS
      ```

      Denotes a request to register a CloudResource entity to a regional CloudInformationService
      (GIS) entity

      See Also:
      :   Constant Field Values


    - #### REQUEST\_REGIONAL\_GIS

      ```
      public static final int REQUEST_REGIONAL_GIS
      ```

      Denotes a request to get a list of other regional GIS entities from the system GIS entity

      See Also:
      :   Constant Field Values


    - #### RESOURCE\_CHARACTERISTICS\_REQUEST

      ```
      public static final int RESOURCE_CHARACTERISTICS_REQUEST
      ```

      Denotes request for grid resource characteristics information. This tag is normally used
      between CloudSim and CloudResource entity.

      See Also:
      :   Constant Field Values


    - #### INFOPKT\_SUBMIT

      ```
      public static final int INFOPKT_SUBMIT
      ```

      This tag is used by an entity to send ping requests

      See Also:
      :   Constant Field Values


    - #### INFOPKT\_RETURN

      ```
      public static final int INFOPKT_RETURN
      ```

      This tag is used to return the ping request back to sender

      See Also:
      :   Constant Field Values


    - #### CLOUDLET\_RETURN

      ```
      public static final int CLOUDLET_RETURN
      ```

      Denotes the return of a Cloudlet back to sender. This tag is normally used by CloudResource
      entity.

      See Also:
      :   Constant Field Values


    - #### CLOUDLET\_SUBMIT

      ```
      public static final int CLOUDLET_SUBMIT
      ```

      Denotes the submission of a Cloudlet. This tag is normally used between CloudSim User and
      CloudResource entity.

      See Also:
      :   Constant Field Values


    - #### CLOUDLET\_SUBMIT\_ACK

      ```
      public static final int CLOUDLET_SUBMIT_ACK
      ```

      Denotes the submission of a Cloudlet with an acknowledgement. This tag is normally used
      between CloudSim User and CloudResource entity.

      See Also:
      :   Constant Field Values


    - #### CLOUDLET\_CANCEL

      ```
      public static final int CLOUDLET_CANCEL
      ```

      Cancels a Cloudlet submitted in the CloudResource entity.

      See Also:
      :   Constant Field Values


    - #### CLOUDLET\_STATUS

      ```
      public static final int CLOUDLET_STATUS
      ```

      Denotes the status of a Cloudlet.

      See Also:
      :   Constant Field Values


    - #### CLOUDLET\_PAUSE

      ```
      public static final int CLOUDLET_PAUSE
      ```

      Pauses a Cloudlet submitted in the CloudResource entity.

      See Also:
      :   Constant Field Values


    - #### CLOUDLET\_PAUSE\_ACK

      ```
      public static final int CLOUDLET_PAUSE_ACK
      ```

      Pauses a Cloudlet submitted in the CloudResource entity with an acknowledgement.

      See Also:
      :   Constant Field Values


    - #### CLOUDLET\_RESUME

      ```
      public static final int CLOUDLET_RESUME
      ```

      Resumes a Cloudlet submitted in the CloudResource entity.

      See Also:
      :   Constant Field Values


    - #### CLOUDLET\_RESUME\_ACK

      ```
      public static final int CLOUDLET_RESUME_ACK
      ```

      Resumes a Cloudlet submitted in the CloudResource entity with an acknowledgement.

      See Also:
      :   Constant Field Values


    - #### CLOUDLET\_MOVE

      ```
      public static final int CLOUDLET_MOVE
      ```

      Moves a Cloudlet to another CloudResource entity.

      See Also:
      :   Constant Field Values


    - #### CLOUDLET\_MOVE\_ACK

      ```
      public static final int CLOUDLET_MOVE_ACK
      ```

      Moves a Cloudlet to another CloudResource entity with an acknowledgement.

      See Also:
      :   Constant Field Values


    - #### VM\_CREATE

      ```
      public static final int VM_CREATE
      ```

      Denotes a request to create a new VM in a Datacentre With acknowledgement information sent by
      the Datacentre

      See Also:
      :   Constant Field Values


    - #### VM\_CREATE\_ACK

      ```
      public static final int VM_CREATE_ACK
      ```

      Denotes a request to create a new VM in a Datacentre With acknowledgement information sent by
      the Datacentre

      See Also:
      :   Constant Field Values


    - #### VM\_DESTROY

      ```
      public static final int VM_DESTROY
      ```

      Denotes a request to destroy a new VM in a Datacentre

      See Also:
      :   Constant Field Values


    - #### VM\_DESTROY\_ACK

      ```
      public static final int VM_DESTROY_ACK
      ```

      Denotes a request to destroy a new VM in a Datacentre

      See Also:
      :   Constant Field Values


    - #### VM\_MIGRATE

      ```
      public static final int VM_MIGRATE
      ```

      Denotes a request to migrate a new VM in a Datacentre

      See Also:
      :   Constant Field Values


    - #### VM\_MIGRATE\_ACK

      ```
      public static final int VM_MIGRATE_ACK
      ```

      Denotes a request to migrate a new VM in a Datacentre With acknowledgement information sent
      by the Datacentre

      See Also:
      :   Constant Field Values


    - #### VM\_DATA\_ADD

      ```
      public static final int VM_DATA_ADD
      ```

      Denotes an event to send a file from a user to a datacenter

      See Also:
      :   Constant Field Values


    - #### VM\_DATA\_ADD\_ACK

      ```
      public static final int VM_DATA_ADD_ACK
      ```

      Denotes an event to send a file from a user to a datacenter

      See Also:
      :   Constant Field Values


    - #### VM\_DATA\_DEL

      ```
      public static final int VM_DATA_DEL
      ```

      Denotes an event to remove a file from a datacenter

      See Also:
      :   Constant Field Values


    - #### VM\_DATA\_DEL\_ACK

      ```
      public static final int VM_DATA_DEL_ACK
      ```

      Denotes an event to remove a file from a datacenter

      See Also:
      :   Constant Field Values


    - #### VM\_DATACENTER\_EVENT

      ```
      public static final int VM_DATACENTER_EVENT
      ```

      Denotes an internal event generated in a PowerDatacenter

      See Also:
      :   Constant Field Values


    - #### VM\_BROKER\_EVENT

      ```
      public static final int VM_BROKER_EVENT
      ```

      Denotes an internal event generated in a Broker

      See Also:
      :   Constant Field Values


    - #### Network\_Event\_UP

      ```
      public static final int Network_Event_UP
      ```

      See Also:
      :   Constant Field Values


    - #### Network\_Event\_send

      ```
      public static final int Network_Event_send
      ```

      See Also:
      :   Constant Field Values


    - #### RESOURCE\_Register

      ```
      public static final int RESOURCE_Register
      ```

      See Also:
      :   Constant Field Values


    - #### Network\_Event\_DOWN

      ```
      public static final int Network_Event_DOWN
      ```

      See Also:
      :   Constant Field Values


    - #### Network\_Event\_Host

      ```
      public static final int Network_Event_Host
      ```

      See Also:
      :   Constant Field Values


    - #### NextCycle

      ```
      public static final int NextCycle
      ```

      See Also:
      :   Constant Field Values


- Overview
- Package
- Class
- Use
- Tree
- Deprecated
- Index
- Help

- Prev Class
- Next Class

- Frames
- No Frames

- All Classes

- Summary:
- Nested |
- Field |
- Constr |
- Method

- Detail:
- Field |
- Constr |
- Method
